# Generative AI as Third Agent: Large Language Models and the Transformation of the Clinician-Patient Relationship

**DOI:** 10.2196/68146

**Published:** 2025-08-11

**Authors:** Hugo de O Campos, Daniel Wolfe, Hongzhou Luan, Ida Sim

**Affiliations:** 1 Strategic Advisory Board, Computational Precision Health, University of California, Berkeley and University of California, San Francisco, 2195 Hearst Ave, Suite 120, Oakland, CA, 94720, United States; 2 Computational Precision Health, University of California, Berkeley and University of California, San Francisco, Berkeley, CA, United States; 3 Department of Medicine, University of California, San Francisco, San Francisco, CA, United States

**Keywords:** artificial intelligence, large language model, generative AI, healthcare, empowerment, patient-clinician relationship, patient engagement

## Abstract

The use of artificial intelligence (AI) in health care has significant implications for patient-clinician interactions. Practical and ethical challenges have emerged with the adoption of large language models (LLMs) that respond to prompts from clinicians, patients, and caregivers. With an emphasis on patient experience, this paper examines the potential of LLMs to act as facilitators, interrupters, or both in patient-clinician relationships. Drawing on our experiences as patient advocates, computer scientists, and physician informaticists working to improve data exchange and patient experience, we examine how LLMs might enhance patient engagement, support triage, and inform clinical decision-making. While affirming LLMs as a tool enabling the rise of the “AI patient,” we also explore concerns surrounding data privacy, algorithmic bias, moral injury, and the erosion of human connection. To help navigate these tensions, we outline a conceptual framework that anticipates the role and impact of LLMs in patient-clinician dynamics and propose key areas for future inquiry. Realizing the potential of LLMs requires careful consideration of which aspects of the patient-clinician relationship must remain distinctly human and why, even when LLMs offer plausible substitutes. This inquiry should draw on ethics and philosophy, aligned with AI imperatives such as patient-centered design and transparency, and shaped through collaboration between technologists, health care providers, and patient communities.

## Introduction

The integration of artificial intelligence (AI) into health care has rapidly transformed various aspects of medical practice, from diagnostics to treatment planning. The emergence of generative AI, particularly large language models (LLMs) that can interact and communicate with humans in a personalized and empathetic way, heralds what some commentators have termed “relational AI,” [1] with LLMs now functioning increasingly like “agents in the clinic” interposing themselves into patient-clinician interactions [2]. It is not yet clear if LLMs will act as facilitators—enhancing communication, supporting decision-making, and strengthening the clinician-patient relationship—or as interrupters, disrupting natural interactions, creating friction, or undermining trust. This paper explores patient and clinician perspectives on that question and proposes areas for inquiry when researching participatory medicine in an AI-enabled clinical relationship.

Patients have long sought medical information and support outside of traditional clinical settings, and since the advent of the internet, have often turned to online resources, listservs, and virtual communities [3]. The emergence of powerful and easily navigated search engines, followed by Web 2.0’s digital and social networking platforms, amplified this trend, offering patients unprecedented access to medical knowledge and peer-to-peer platforms for sharing personal medical information and clinical experiences [4]. The rise of internet use for health information and self-diagnosis fueled a movement of e-patients [5] even as medical professionals raised concerns regarding the impact of “Dr. Google” on patient-clinician relations. The ship is sailed: as many as half of Americans seek health care information online for themselves or others, without evidence of negative effects on health outcomes or the patient-clinician relationship [3,6,7].

Legislative and technological developments have given new data and scope to the e-patient movement. More than 1 in 3 Americans now own a wearable or portable device that collects information about steps, sleep, blood pressure, heart rate, or other indicators of health or fitness [8]. The 21st Century Cures Act has required health systems to adopt information standards allowing data export and interoperability with other systems and mandated that patients have electronic access to their health information at no cost [9]. Advocates from the OpenNotes movement, which promotes trust-building through the sharing of medical records between health care providers and patients, have heralded these developments as key enablers of a shift to democratized, person-centric, and participatory health care [10,11]. If, as the e-patient movement suggests, data is power [12], then new types of data—and new ways of collecting and accessing it—can further empower patients. LLMs that allow patients and providers to organize health information, draw insights from it, and increasingly engage in iterative “prompting” with personal health data add a new layer to this already intricate landscape.

Building on the framing of LLMs as a “relational” technology, this paper focuses on their impact on clinician-patient relations. LLMs have been simultaneously hailed as a “turning point in patient power” [13] and as an unreliable, inconsistent, and unaccountable tool that is dangerous for medical use [14]. The degree to which LLM use will be acknowledged during a clinical encounter, and whether or not LLMs are a facilitator of or impediment to therapeutic alliance, are open questions. Writing from our perspectives as patient advocates, computer scientists, and physician-informaticists, we explore various potential roles for LLMs in the clinical exchange. We propose areas of research aimed at better understanding current attitudes toward and uses of LLMs, while moving us toward a collaborative use that could enrich therapeutic alliance and health outcomes.

## Patient and Personal Care Team: The Rise of the AI Patient

Even before LLMs, patients regarded AI use in health care with ambivalence. Six in ten said they would be uncomfortable with their provider relying on AI in providing their care. A nearly equal number thought that using AI to diagnose disease or recommend treatment would make relations with clinicians worse. At the same time, a majority believed racial bias would be decreased if AI were used more to do things such as diagnose disease and recommend treatments for patients [15].

LLMs, and the associated ability of patients to move from passive objects of AI to more active users, may change the calculus. We join those patient advocates who see LLMs as a new and important tool in health care and self-care, particularly in addressing the gaps in access and communication that often plague the current health care system. Chat interfaces like OpenAI’s ChatGPT have opened options previously unavailable to the e-patient, lowering the technology and language literacy barrier, allowing patients to ask questions in multiple languages, generating responses tailored to different audiences and responsive to requests for clarification or further elucidation. By synthesizing complex, highly technical health-related literature or multiple examination results into understandable summaries for a range of educational levels, LLMs have become valuable tools for participatory medicine. For patients and their families, these technologies facilitate a fundamental and empowering shift in the flow of information, moving from patients to doctors rather than the other way around [13]. If electronic medical records and the internet enabled the rise of the e-patient, LLMs are now driving the rise of the AI patient [16].

As a caretaker for his elderly father and a patient with a genetic heart condition, one of us (HdOC) relies on LLMs for various tasks, including preparing for appointments, organizing health information, weighing the pros and cons of different medical interventions, and summarizing medical notes for family members not fluent in English. HdOC’s recent leveraging of LLMs to navigate his father’s complex medical needs demonstrates the technology’s potential to empower informed patients and bridge information gaps (Textbox 1). More generally, LLMs are already being used by patients and by the families and caregivers integral to their care to simplify and improve understanding of informed consent forms, to parse complicated communications from insurance companies or a medical note, to better understand laboratory notes, and to translate any and all of the above into different languages [17,18].

While all patients have the potential to increase their sense of agency and engagement in their health care, not all have the technological literacy to use LLMs to advance participatory medicine. AI may thus exacerbate existing health disparities and create a digital divide between those with internet access and those without, and between those who have or who lack the skills and resources to use AI tools effectively. Former Google CEO Eric Schmidt, speaking to Stanford students at a 2024 forum on AI’s likely impact on global development, offered a prognosis relevant here: “the rich get richer, and the poor do the best they can” [19]. For example, the size of the “context window” that determines the amount of information that an LLM can take in is expected shortly to grow to more than a million tokens, or the equivalent of 750,000 words. This massive expansion, a 45-fold increase from earlier models such as GPT 3.5, will mean that those who can pay for premium LLM services will receive more personalized and contextualized answers. Those relegated to smaller-capacity, free LLM services will not. Even use of freely available tools will require understanding of multiple dimensions: English proficiency, medical literacy, numeracy, and technical and critical thinking skills to help them make informed decisions in an increasingly AI-mediated health care landscape [20].

Textbox 1. **Case example.**

One of us (HdOC) faced a challenging situation when his older adult father developed a severe pruritic rash, and the earliest available dermatologist appointment was months away. Drawing from his experience using large language models (LLMs) for his own health care, HdOC turned to various publicly available chatbots, including Anthropic’s Claude 3 Opus, Perplexity AI’s Perplexity, and OpenAI’s GPT-4o, for counsel on his father’s condition. He meticulously collected his father’s medical records by downloading clinical notes from past clinical encounters and accessing blood test results (with permission) through his father’s electronic patient record portal. Armed with these records, including laboratory results, recent clinical notes, and photos of the rash, HdOC then used multiple LLMs to analyze these inputs. The models provided differential diagnoses, recommended actions, and identified a potential link between the rash and his father’s underlying chronic kidney disease.

HdOC then developed a multipronged treatment plan based on LLM recommendations. The plan included strict dietary adjustments to manage kidney function, reducing shower frequency and temperature to prevent skin dryness, aggressive moisturizing with fragrance-free products, and the application of topical corticosteroids to control itching and inflammation. HdOC also used the LLMs to translate this information into Portuguese, ensuring that HdOC’s father could understand the proposed treatment plan and participate in the decision-making process.

By comparing outputs from different LLMs and validating AI-suggested interventions through online searches and email correspondence with medical professionals, HdOC implemented a care plan that significantly improved the rash within 10 days. By the time the appointment with the dermatologist arrived, the rash had mostly cleared up. Strategic use of the tools enabled an approach that transformed the traditional patient-clinician dynamic into a more equal partnership, correcting power and information asymmetry, and ultimately leading to better outcomes and enhanced patient satisfaction.

Prompts and outputs from LLMs are included in Multimedia Appendix 1.

While the use of an LLM as an ally or “doctor in your pocket” holds great potential, integrating LLM insights into the patient-clinician relationship remains a challenge. Just as the e-patient movement emphasized patient control over “our data,” AI patients are likely to support LLMs for personal health use but may be wary of their adoption by institutions, health systems, or commercial entities. Comfort about when or if LLMs are used in the diagnostic and care pathway may vary by patient and condition: surveys of patients asked to consider AI use to augment or replace physician input in the years before LLM availability, for example, found significant differences in concerns about privacy and AI-assisted diagnosis among those with chronic or acute conditions, as well as variation in understanding of AI function by age and demographics [21]. Patients with rare diseases and their family members today, for example, are significantly more likely to use LLMs for health assistance than other patients [22]—we do yet know how this impacts their interactions with clinicians, or if it will positively impact their care. Diagnostic errors with general purpose LLMs such as GPT (OpenAI), Llama (Meta AI), or Gemini (Google LLC) are a particular area of concern—multiple studies show that while these models can answer examination questions or analyze clinical vignettes correctly, they often produce diagnostic conclusions or responses at variance with clinician recommendations when confronted with real-life, “noisy” medical data, and reproduce racial or gender biases and stereotypes adversely impacting diagnosis [23-27].

Usage transparency—knowing when and how LLMs are being used by any part of the health care system—is also likely to be key to patient trust. Patients discern whether medical professionals or health systems deem them worthy of enough respect to disclose when AI has been deployed in their care and to inform them of potential limitations [28]. A patient at a recent advocacy forum shared her experience with a clinic representative named Jennifer, with whom she had been messaging about medication refills and scheduling an appointment. Jennifer was helpful, kind, and friendly, even engaging in casual conversation about personal topics. But when the patient arrived for her appointment and asked to say hello to Jennifer, she was surprised to learn that Jennifer was a chatbot—not a real person (personal communication, 2024). Many patients express discomfort when an LLM is used to replace a genuine human connection. But is the discomfort arising from being misled, or does it stem from deeper existential concerns about forming relationships with a nonhuman entity? Can these qualms be overcome? And should they be?

## Keyboard Liberation or Loss of Human Connection?

### Promise and Pitfalls

For many patients and clinicians, the most immediate use case for LLMs is what Eric Topol famously termed “keyboard liberation,” [29] reducing time spent feeding information into electronic health records (EHRs) and increasing opportunities for interaction. LLM-driven scribing systems, which listen to patient-clinician exchanges and automatically generate large parts of clinical notes and after-visit summaries, are increasingly deployed in well-resourced, AI-capable health care systems [30]. Early reports are that both patients and clinicians feel more connected when the clinician can shift attention from the keyboard [31]. By taking over rote administrative tasks and allowing clinicians to shift their focus from screens back to patients, the best-case scenario is that LLMs will free up clinicians to practice “at the top of their license,” reducing clinician burnout and improving patient experience [32,33].

The same advances that promise liberation, however, may also bring unanticipated and undesired shifts in roles. For nearly a decade, analysts have debated whether various physician roles—from radiologists to primary care providers—will be needed at all in an AI-enabled future, or whether replacement with AI-enabled avatars could reduce the burden and increase health care delivery [34,35]. Current LLMs remain vulnerable to hallucinations, errors of fact or reasoning that make the elimination of a human in the loop inadvisable. Performance can be improved through human correction (eg, reinforcement learning through human feedback), and through the fine-tuning of smaller, more health care–focused models by supplementing their built-in knowledge with a connection to external medical databases and peer-reviewed literature. Whether this method—known as retrieval-augmented generation—will improve LLMs to allow for unsupervised diagnosis remains uncertain.

The risk that LLMs will eclipse humans in the clinical encounter is a concern even when clinicians are present. Analysts have warned that as health systems increase their use of LLMs, human clinical skills may degrade over time, particularly as LLMs ingest AI-generated data for training, creating a self-referential and increasingly machine-driven learning loop [14]. Automation bias—the belief that the machine-generated insights are more authoritative than they actually are—is another concern raised by those analyzing the potential impacts of physician use of LLM-generated notes in the EHR [36]. The same bias may apply to patients using LLMs to organize and analyze medical information.

Finally, LLM recommendations may add moral injury to the clinical encounter. Managed care has for some time required physicians to play a dual and conflicted role in the health system, tasked both with protecting patient well-being and achieving cost containment or other health system priorities [37]. It is easy to imagine LLMs trained by payors or health systems mandating that clinicians adhere to algorithmically determined actions even when these conflict with their clinical judgment on what is best for patient health.

### Strengthening the Human in the Clinical Exchange

LLM use has sharpened longstanding questions about which qualities in care are considered essentially human, and how these impact the patient-clinician relationship. On the one hand, LLMs have highlighted the patient view that human clinicians might benefit from their own “fine-tuning”: in a study comparing physician and AI chatbot responses to questions on a public forum, patients rated the AI responses as more empathetic [38]. However, critics argue that such expressions amount to “artificial empathy”—a superficial simulation rather than a genuine recognition of patient worry or suffering [39]. Just as selecting the correct answer on a multiple-choice medical examination cannot replace a seasoned clinician’s intuition or ability to recognize subtle patterns [40], an empathetic-sounding reply does not equal the deeper understanding of a patient’s distress—or sensitivity to the moral and cultural values that shape appropriate response—that defines authentic human empathy in care.

What do patients value? Busch et al [41] conducted a meta-analysis of studies examining what patients and caregivers regarded as central to humanistic exchange in clinical encounters. They found that a majority highlighted 6 elements. Each raises questions about whether LLMs, regardless of their command of medical facts, will be able to reproduce these elements or fall short (Textbox 2).

Textbox 2. **Key elements of humanistic care: the patient’s view.**

While competence in diagnosis and treatment is a key concern, many other factors also determine what patients and caregivers value in care. A meta-analysis by Busch et al [41] found that a majority identified the 6 elements below as key to humanistic care. Each suggests questions about how LLM use might facilitate or impede them.

**1. Empathy.** This extends beyond the clinical encounter to include genuine, emotionally engaged awareness of patient or caregiver experience outside the clinic, and clinician openness in learning more about the complexity of the patient’s point of view [42].

**2. Respect for patients’ (and caregivers’) dignity, uniqueness, individuality, and humanity.** In addition to respectful care delivery in the clinical exchange, this includes attention to prevention and treatment in the context of the patient’s life course, and a focus on individuals’ (and caregivers’) preferences and values [41].

**3. Relationship bonding.** Additionally referred to as therapeutic alliance, this is a shared sense between clinician and patient that affirms the collaborative nature of the relationship, shared emotional bond, and agreement on treatment goals and tasks [43].

**4. Respect for patient autonomy and involvement.** This includes creation of an environment where patients (and their caregivers) feel safe expressing their concerns, or disagreeing with or exploring alternatives to clinical recommendations [44]

**5. Communication.** In addition to clear verbal communication, this includes nonverbal communication—tone of voice, eye contact, and facial expressions, as well as things such as examination room characteristics, touch, interpersonal distance, and clinician clothing, gestures, and posture [45].

**6. Patience and commitment.** While these are difficult to define, both include care that allows time and interest in patient engagement during the clinic visit and beyond, without patients feeling rushed or dismissed and with a sense of clinician interest in patient progress over time [41].

## LLM as Third Agent in the Clinical Encounter

###  Evolution of the Doctor-Patient Relationship

The doctor-patient relationship has historically been seen as the bedrock of medicine: even now, while the patient may have family caregivers and the clinician may practice within a clinical care team or health system, the direct, one-on-one human connection between patient and clinician remains an ideal. In this model, the doctor is a trusted confidante whose role as a medical and even moral adviser to the patient has moved some analysts to describe the doctor-patient relationship as similar to that of a parent and child [46,47].

Medical historians note that this idealized view of the family doctor was already out of date for much of the 20th century, even as it remained the dominant cultural narrative [48]. By the 1990s, the hallmarks of that relationship—physicians as carers for the whole family, and with freedom to act as they saw fit to safeguard patient health—were largely no longer in place. The growth of managed care, capitation, and other health system changes contributed to this shift. Changes in patient self-concept and advances in digital technology—including online medical information platforms, increased patient access to their own electronic medical records, and the overall movement for patient self-advocacy—further accelerated the transformation [37,49-51].

### New Trilateral Framework

LLMs now introduce a new, third agent, shaping communication, understanding, and connection between patients and caregivers and their clinicians. We present a framework for describing and analyzing this new interaction, in which patients (and their surrogates) as well as clinicians at all skill levels avail themselves of the power of LLMs to review background information, secure diagnostic or therapeutic assistance, or navigate choices.

**Figure 1.**
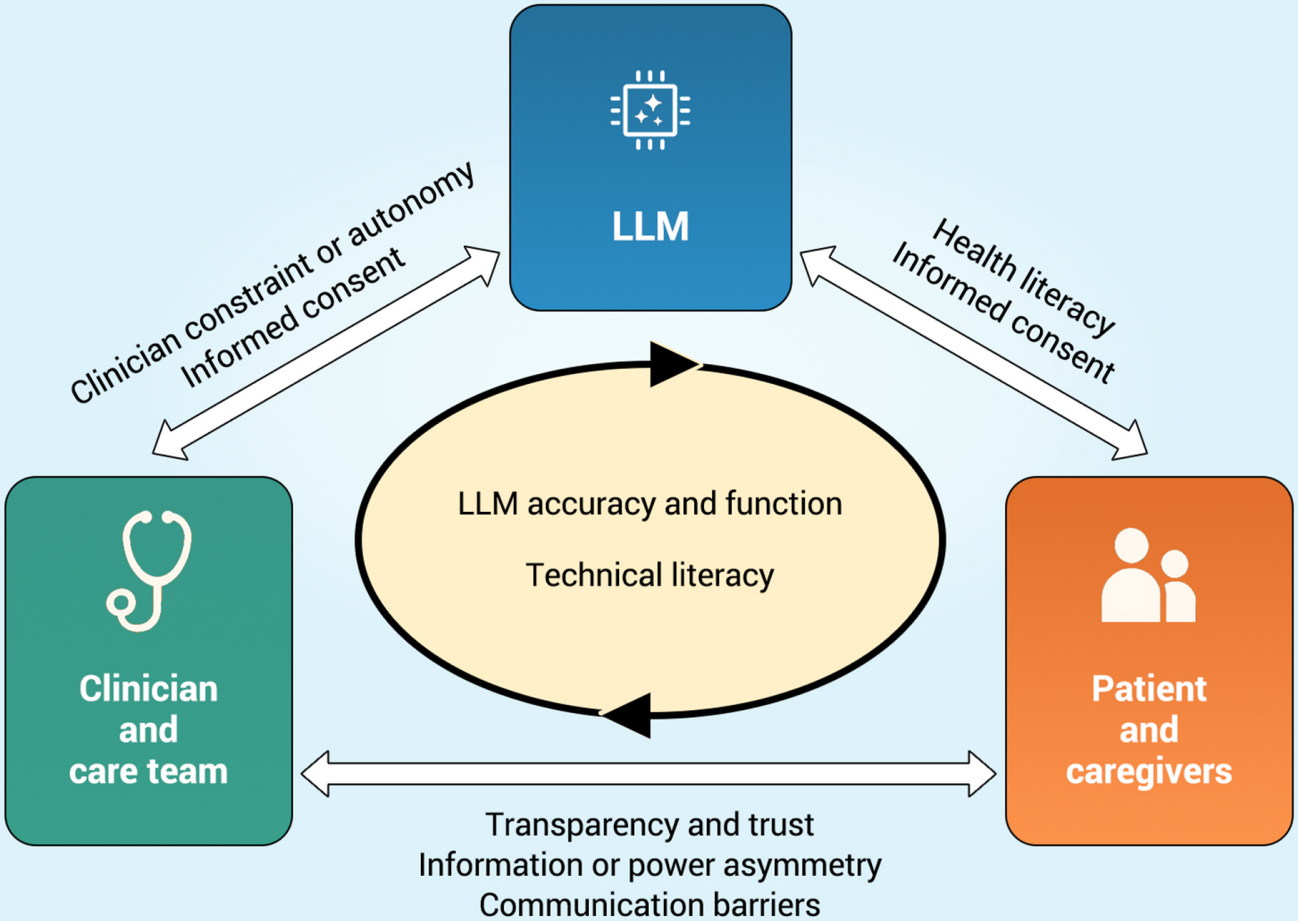
Trilateral interaction framework—LLMs as a third agent in the patient-clinician relationship, and factors mediating exchange. LLM: large language model.

Figure 1 illustrates the current state of this new trilateral (3-sided) interaction. Each “corner” of the triangle represents an actor participating in the exchange of information. Two corners are inhabited by human actors: patients and their caregivers, and clinicians and clinical care teams. The third corner is now inhabited by LLMs, which are used by both clinicians and patients to generate content, analysis, and recommendations. Between these corners are “edges” representing interactions between humans and machines as well as between human actors.

Accuracy of LLMs and the knowledge required to use them are factors that impact all dynamics, and so are at the center of the diagram. Other elements mediating the nature of the interactions are noted along each edge of the clinical exchange. For clinicians, LLM use may be constrained by the permissions or restrictions imposed by the system in which they work. For patients and caregivers, health literacy—including the ability to detect likely hallucinations or assess the reliability of cited sources—will impact both the use and usefulness of LLMs. Factors that have shaped clinician-patient relations since well before the LLMs remain relevant and are noted underneath the arrow indicating interaction between physician and patient, including power and information asymmetries, trust, and the quality of communication.

As LLMs currently play a limited role in generating direct communication between physician and patient, nothing links the LLM directly to the arrow representing doctor-patient exchange. While some health systems are deploying AI-generated “smart replies” to patients, these are generally only for routine matters such as scheduling of appointments, prescription refills, or the like, and only after clinician review and approval. A recent study of such AI-generated replies has found that clinicians deemed only 20% of drafts usable [52]. For their part, some “AI patients” have also begun to use LLMs to compose or clarify communications to clinicians, or to help raise the possibility of new diagnoses or course of treatment [53]. A paramount concern is how these trilateral interactions will impact patients’ and clinicians’ sense of their own agency and trust in other humans, and in the overall health care system.

### LLM as Interrupter?

LLMs can expand capacity across a range of health care actors, delivering new knowledge, predictive insights, or recommendations to patients and their families, as well as to physicians, nurses, physician assistants, and pharmacists. As noted, although the multilingual translation capabilities of LLM-based chatbots have not been fully evaluated for medical accuracy or tested with non-English prompts, they are likely to improve comprehension in families where patients or caregivers are not native speakers.

**Figure 2.**
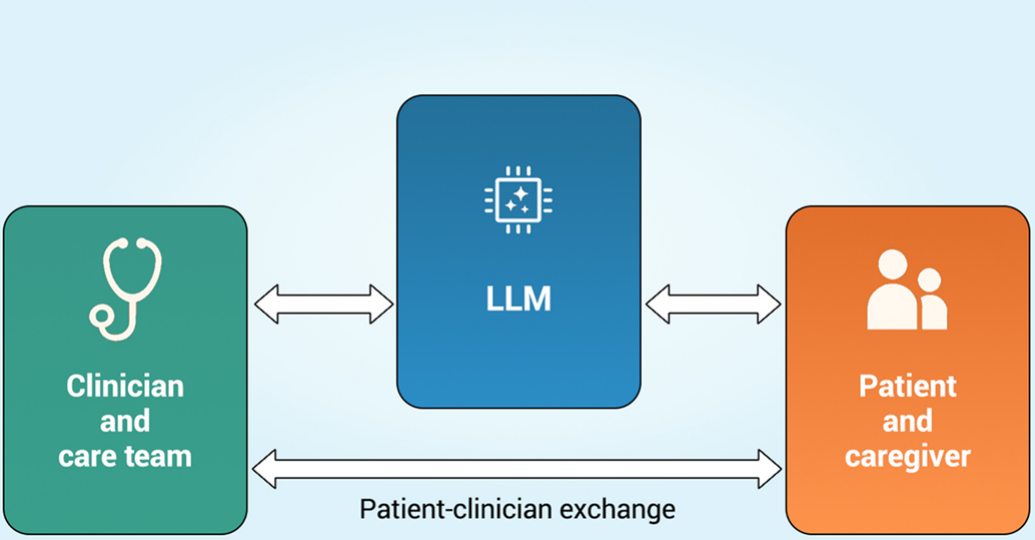
LLM as an interrupter in clinician-patient interaction. LLM: large language model.

This generative power, however, may diminish or interrupt humanistic exchange. As LLMs improve their capacity to generate communications without human supervision or refinement, it is not difficult to imagine health systems using them as a substitute for, rather than a facilitator of, human-to-human interactions (Figure 2). In some cases, the AI models—trained by health systems—may have goals that differ from those of doctors, leading to recommendations that prioritize cost saving over care, or that are insensitive to patients’ moral or cultural values. Patient use of smartphone photos and LLMs for self-diagnosis of dermatological conditions, or use of ChatGPT to diagnose cause of stomachache or cough without consultation with medical professionals [6,54], represents present-day scenarios where the LLM, functioning as a “doctor in your pocket,” is less a facilitator of exchange between patient and clinician than its interrupter [6,55].

Reductions in human-to-human exchange risks loss of the therapeutic alliance, sense of shared purpose, respect, and connectedness that defines humanistic care. As noted in the anecdote above, where a patient had been interacting with an LLM without knowing it, this framework also suggests the need for new ethics governing trilateral, AI-involved medical exchange.

The health impact of this blurring of boundaries between human and machine and the diminution of human interaction is not yet clear. LLMs are evolving, and their power to simulate human relations raises the question of whether machine-generated therapeutic alliances might be as “good” in some practical sense as those created through human interaction. Might generative AI someday reproduce all the qualities in Textbox 2? Older adult Japanese patients have experienced decreased loneliness with therapeutic robot pets, such as small mechanical seals [56], and children with developmental difficulties have found benefit from robot playmates [57]. Generative AI may possess similar or greater powers of comfort. At the same time, as psychiatrist and medical anthropologist Kleinman [58] reminds us, caregiving is relational and reciprocal, including both a range of physical acts—touch, embrace, lifting, steadying, toileting, and more—as well as the way we look at another human being, receive their gaze, experience a quality of voice or physical presence as an expression of solidarity and moral support. For Kleinman and countless patients and families, these essential elements of human care had become mechanized and inauthentic in much of modern health care even before the advent of the LLM. The ineffably human dimensions of care—moments of connection, physical presence, deep empathy, and moral solidarity—are unlikely to be replicated by even the most sophisticated language models, no matter how well-prompted or finely tuned.

### Longer Term: Agentic AI as Ally or Facilitator?

**Figure 3.**
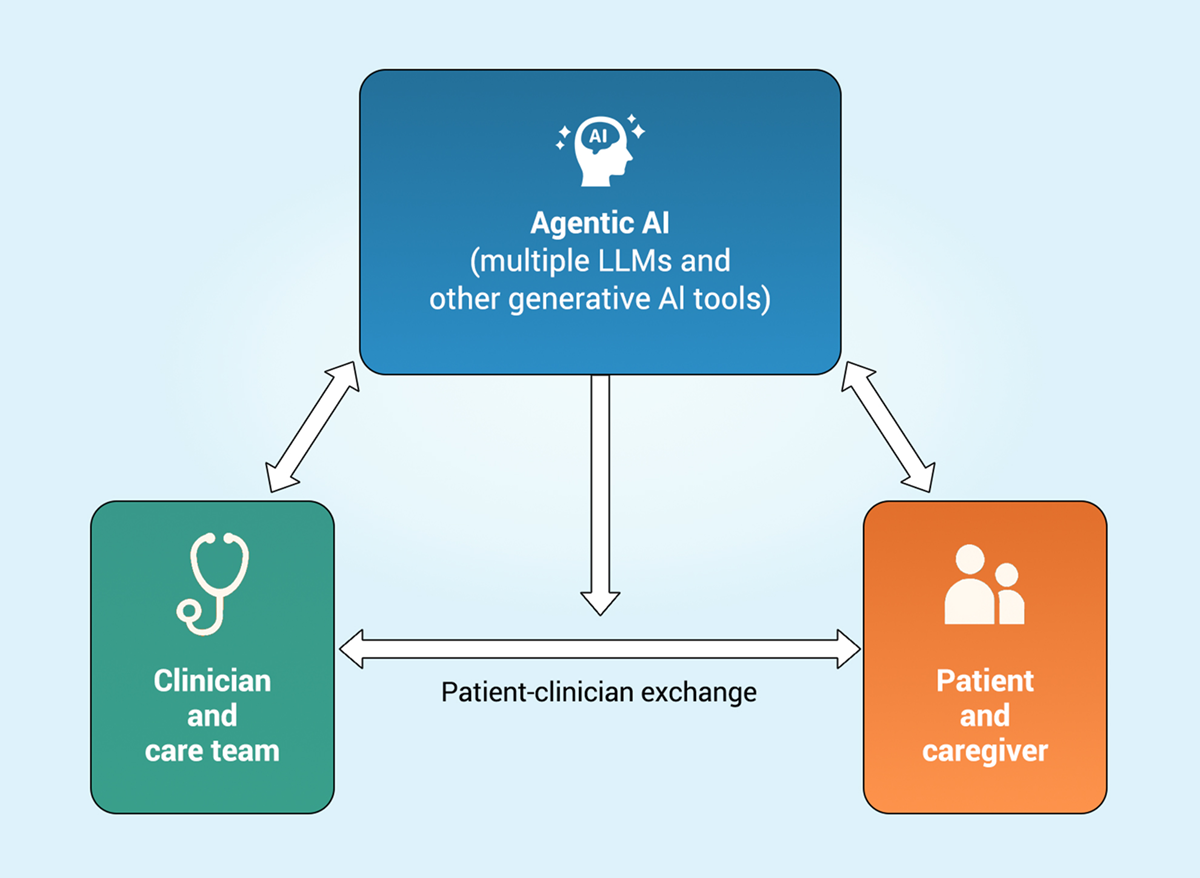
Agentic AI as a facilitator between clinician and patient. While clinicians and patients both use agentic AI, humanistic exchange remains robust. AI: artificial intelligence; LLM: large language model.

Generative AI is already evolving beyond prompted responses from chatbots to enable what is termed “agentic AI”—systems capable of initiating autonomous action in the virtual and physical world, potentially serving as loyal assistants while preserving human agency. In this scenario, AI can become allies for clinicians, patients, and their respective care teams, facilitating rather than replacing their essential partnership (Figure 3). Agentic AI is assisting in mediating communication but is not eclipsing human exchange. Clinicians and computer scientists working with them have already begun discussion of agentic “AI teammates”—that is, tools to enhance decision-making capacity, parsing clinical records, initiating routine tasks such as prior authorizations, assessing medication interactions, and recommending treatment regimens or preventive strategies tailored to individual patient needs [59,60]. These scenarios, however, have tended to omit attention to patients and their caregivers, for whom agentic AI could similarly serve as a navigator and advocate, organizing clinical records and synthesizing data and medical knowledge, illuminating health determinants and advisable courses of action.

Agentic AI will represent an evolution beyond current LLM capabilities, joining a range of AI tools to initiate actions autonomously according to tailored parameters. Unlike today’s LLMs, which primarily generate text in response to user queries, agentic AI systems will learn from patterns of use, anticipate needs, and act proactively. For example, they might autonomously organize clinical data, flag potential drug interactions, or offer unprompted suggestions or questions for patients and providers to consider in recommending or adjusting treatment regimens, preventive strategies, or diagnoses. AI agents may also autonomously carry out actions such as typing into a computer or clicking on buttons (called “computer use”) [61]—for example, submitting a request for a prescription renewal, contesting the denial of an insurance claim, or changing the alert settings for a continuous glucose monitor.

Agentic AI will also help patients advocate for themselves, recommending strategies to increase patient independence or preferred approaches to address health challenges, while identifying attempts by health care or insurance systems to limit patient choice or impose unwanted treatment pressures. For physicians, AI agents could similarly be tailored or trained to their clinical preferences and style, drawing on lessons from experiences with particular patients and improving their ability to tailor both their communication and approaches to care. In this vision, both patients and clinicians may come to view their AI tools, implicitly or explicitly, as “theirs,” that is, trained by them to serve their specific interests. The embedding of agentic AI in the clinical relationship could nonetheless align patient and clinician in working toward shared outcomes and improved health.

## Generative AI for Participatory Medicine: Areas for Inquiry

### LLM Use, Function, and Safety in the Clinician-Patient Exchange

The newness of generative AI as a third agent in the clinician-patient relationship raises multiple questions, as yet largely unanswered, on whether these new models are fit for purpose. How will patients or clinicians use “their” AI to organize information before, during, or after a visit? How well or safely will the tools perform the tasks required? How will use vary? While answers are likely to change rapidly with field advances, key research directions can include:

### Human Use of General-Purpose LLM Chatbots In Health and Health Care

Rapid uptake of these tools, and rapidly growing capacity to ingest images, PDFs, and increasing numbers of words, make understanding current and potential uses by patients and clinicians critical to understanding likely trajectories for off-the-shelf LLMs and those fine-tuned for health care applications. Key questions include patient assessment of intelligibility of LLM responses, including for those with different English language or educational levels, technical literacy, and assessment of accuracy of responses to patient queries, etc. What is the impact of biases or style embedded in particular LLMs, and how might those interact with patient need or preference? As with model training, research on LLMs as third agents will require diverse datasets and participants, including patients of different races, ethnicities, or gender identities, levels of English proficiency and education, health conditions, comfort with technology, and internet access, as well as varying preferences for communication and recommendations in health care.

### Customization and Optimization of LLMs For Specific Clinical Purposes (eg, Diagnosis and Care Navigation) for Specific Users, Whether Clinicians, Patients, Or Caregivers

 Research should examine both proposed solutions and methods development—including but not limited to pretraining of focused foundation models, incorporation of multiple forms of data (images, sensor data, or EHRs), quantification of uncertainty for estimates produced, and methods for fine-tuning and debiasing. Optimization work, with particular attention to retrieval-augmented generation, is already being carried out at a rapid pace in industry and academia, with industry likely to progress more rapidly given its disproportionate access to large-scale compute capacity. This raises collateral but related research questions on tensions or concordance between patient needs and health care market incentives, regulatory requirements in marketing and labelling of LLM applications to health, potential impact of use agreements between particular LLM or EHR vendors, and differentials in use or constraints to use varying by health care system (public vs private), budget, geography, etc.

### Mediators in Generative AI Use: Health Literacy, Trust and Transparency, Clinician-Patient Power Dynamics and Beyond

### Research Priorities

Participatory medicine emphasizes reducing information asymmetries and increasing trust as key to enabling patients and caregivers to become more effective partners in the clinical setting. The factors mediating relations between the agents in the trilateral framework of patient, clinician, and LLM in Figure 1 raise a range of research questions beyond assessment of the function of off-the-shelf or fine-tuned LLMs. Priorities for this research include approaches to health and technical literacy, informed consent and ethical use, transparency and trust, removal of communication barriers and gains in efficiency, clinician constraint and autonomy, and value of the human in care. 

#### Approaches to Health and Technical Literacy

How does patient empowerment increase numeracy, critical thinking, or effective use of one or more LLM chatbots? Current versions of off-the-shelf LLMs do not cite sources or rank them by accuracy, leaving patients to distinguish between hallucination and reality, or between more and less authoritative sources of information. What strategies can be used to increase patient comfort with LLM use, comparison between models, and the ability to distinguish between recommendations based on low or high strength of evidence or rigor of sources. Literacy in data import or access is also variable—while all patients now have the potential to access medical records from across multiple health systems, knowing how to do that and how to feed results to LLMs will determine whether or not these tools significantly alter information asymmetry.

#### Informed Consent and Ethical Use

 For patients, what is understood regarding privacy, informed consent, or the ability to opt out when their health data is used by health systems to train LLMs? When they upload their personal medical data via a chatbot, what do they understand about the uses that can be made of that information? ChatGPT is now used, with apparent success, to simplify informed consent forms for both clinical research and before surgical procedures [62]. But how informed are clinicians themselves about the potential use of their practice patterns to train generative AI, or about the training data or testing of LLMs in a clinical context? Guidelines urging AI that is “FAVES”—”fair, accurate, verifiable, effective, and safe”—or calls for centralized laboratories to evaluate health AI safety and effectiveness may be insufficient either to address such questions as the impact of LLMs drift in function over time, or to assess impacts of LLM use on patient or workforce morale at point of care.

#### Transparency and Trust

 We cite the instance above of patient disappointment upon discovering that her interlocutor was in fact a chatbot rather than a human provider. What is the impact of disclosure by patients, physicians, and health systems of LLM use, or of not disclosing the use at all? One study of “smart replies” outside the health domain found that when participants think their communication partner is using AI-generated responses, they perceive them as “less cooperative” or “affiliative.” When AI’s role in authoring the responses was unknown, those receiving them judged their interlocutors to be more cooperative collaborators [54]. Whether or how patients and physicians reveal use of AI assistance, under what circumstances this is judged a positive or negative, and whether perception on the benefits of LLM use varies by patient or physician type, health condition, or health system are all questions of interest. Measurement of trust and partnership needs to begin with the design stage, with inclusion of patients in the cocreation of research methods and aims central to research success [51].

#### Removal of Communication Barriers and Gains in Efficiency

 With physician shortages projected to reach 86,000 in the United States within the next decade [63], how will LLM use allow existing clinicians to do more, or reduce the need for exchange with patients? How might patient use of these tools, or even of AI-generated summaries of key data points (including from different health systems, from wearable data inaccessible via the EHR, etc.) speed or improve communication with clinicians?

#### Clinician Constraint and Autonomy

 Clinician priorities and commitment to care are not necessarily aligned with health system priorities. Whether generative AI’s potential in the health system is realized depends in part on whether the cost of deploying and maintaining the innovations is offset by increased incoming revenue or decreases in the expense of replacing burned-out clinicians. Regulatory or liability concerns may also constrain health systems or physicians, leaving patients freer than clinicians in some instances to explore LLM-generated insights about their conditions. How or if limits on clinician autonomy impact the use of generative AI, physician sense of self-efficacy or cognitive load, and patient experience are all research questions of interest.

#### Value of the Human in Care

 The importance to patients of human caring in health care in the age of generative AI, or the degree to which clinicians value their role or humanistic exchange as integral to a process of caregiving, is not yet known. This may vary by patient, condition, specialty or primary care, or health system and depend on patient access to or help from other human actors, including family and other service providers. As electronic, LLM-generated communication between patients and clinicians grows, or as patients or physicians turn to avatars or AI agents to represent them, the question of how much human exchange is needed and what is essentially human about such exchange will become increasingly central.

## Conclusion

The LLM is a new change agent in the health care dynamic, and one with transformative potential for patients and clinicians. Clear-eyed research into both the function and use of LLMs can help bend the arc of that change toward mutual benefit. The key lies not in advancing LLMs as a replacement for clinician-patient interaction, but as a tool to augment it. By conceptualizing something closer to “assistive intelligence,” we can leverage LLMs to enhance and facilitate human connections and collaboration, supporting sound clinical decision-making and improved communication. For patients, in particular, LLMs represent a powerful corrective to power and knowledge imbalances and may lead to a more effective clinician-patient partnership.

Understanding the impact of LLMs and agentic AI on clinician-patient relations will require social science and computer science, qualitative research, as well as quantitative analytics and software engineering. While a focus on clinician-patient interactions is insufficient to address the multiple incentives and forces that underlie the American health care system, understanding the dynamics of those interactions—and acting to design, train, and use LLMs in ways that reinforce humanistic collaboration—is possible and necessary. Adhering to the principles of engagement, cocreation, and ethics that have emerged from patient movements can create a future where AI serves as a facilitator of the communication and connection at the heart of human-centered and effective care.

## Supplementary material

10.2196/68146 Multimedia Appendix 1 Prompts and outputs from LLMs. LLM: large language model.

## Abbreviations:

AI: artificial intelligence
EHR: electronic health record
LLM: large language model
