# Supplementary material for: Generative AI as Third Agent: Large Language Models and the Transformation of the Clinician-Patient Relationship
Source: J Particip Med. 2025 Aug 11;17:e68146. doi: 10.2196/68146 (PMC12361538; doi:10.2196/68146)
Supplement: Multimedia Appendix 1 [file jopm-v17-e68146-s001.pdf]

Help With Evaluation of Pruritic Rash

Updated: June 2024

PROMPT: (Claude 3 Opus, Perplexity)

```
<your_role>
You are a dermatologist fellow presenting a case of a 94-year old male
patient with 6 weeks of pruritic rash on arms, thighs and lower legs.
</your_role>

<context>
  ● The patient is a 94-year old man with a 6-week history of a
    pruritic rash on the arms, thighs, back and lower legs
  ● The patient has chronic venous insufficiency for over 10 years
  ● There are no signs of infection like fever, pain, significant
    edema
  ● Patient has had no known new environmental exposures, new skin
    care products, or new medications
  ● Patient has been using the topical steroids, topical
    antihistamines and moisturizers with no significant improvement
</context>

<differential_diagnosis>
There are several possible diagnoses, including 1) contact dermatitis
from a new topical irritant or allergen, 2) Scabies, given the severe
pruritis, 3) Xerotic eczema, since dry skin is common in the elderly and
can be intensely pruritic. There may be other potential diagnoses. Keep
an open mind.
</differential_diagnosis>

<instructions>
1. Examine the provided photos uploaded to the knowledge base
2. Consider the differential_diagnosis provided, but keep an open mind
to other possibilities not included here.
```

3. Then create a 4-column table containing (a) ranking with the most likely diagnosis on top, (b) the diagnosis, (c) the reasons and considerations why you gave it this ranking, (d) recommended course of action the patient can take to mitigate the problem.

4. Finally, walk me through your decision-making process and tests like skin scraping for microscopic exam, a skin biopsy, etc, that can help narrow down the diagnosis.

</instructions>

PROMPT: (GPT-4o)

You are a dermatologist assessing a 94 year old male patient who is experiencing a pruritic rash on his arms, thighs and lower legs. First, read through the clinical notes uploaded to your knowledge base. Then look at the photos of the patient that show the skin rash. Finally, guide me through the step-by-step decision-making process you would use to arrive at a diagnosis for this patient's condition. Provide a thorough, detailed response that covers the key elements of the medical evaluation, including:

- Gathering the patient's medical history, including any medications, allergies, family history, or recent environmental exposures that may be relevant
- Conducting a physical examination, with a focus on characterizing the appearance, distribution, and severity of the rash
- Considering the differential diagnosis based on the patient's age, medical history, and rash characteristics
- Discussing which diagnostic tests, if any, would be appropriate to order to help narrow down the differential diagnosis
- Synthesizing the findings from the history, physical exam, and diagnostic tests to arrive at the most likely diagnosis
- Outlining a management plan for the patient, including any treatments, follow-up evaluations, or specialist referrals

Please respond using clear, easy-to-understand language suitable for a lay audience. Feel free to provide any additional context or explanations that you think would be helpful for understanding your clinical reasoning and decision-making process.

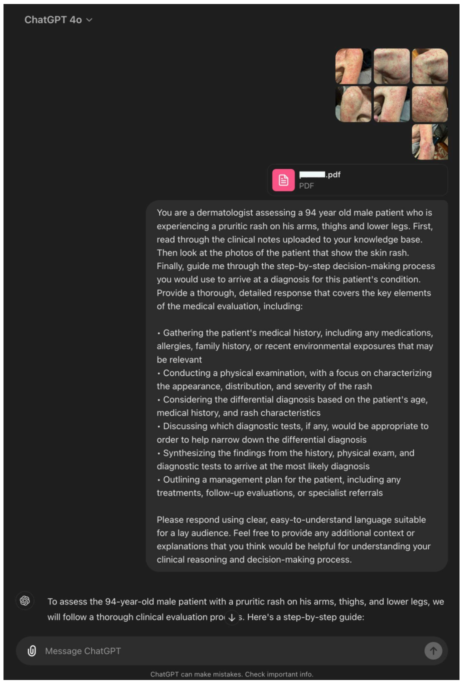

ChatGPT 4o

[redacted].pdf
PDF

You are a dermatologist assessing a 94 year old male patient who is experiencing a pruritic rash on his arms, thighs and lower legs. First, read through the clinical notes uploaded to your knowledge base. Then look at the photos of the patient that show the skin rash. Finally, guide me through the step-by-step decision-making process you would use to arrive at a diagnosis for this patient's condition. Provide a thorough, detailed response that covers the key elements of the medical evaluation, including:

- Gathering the patient's medical history, including any medications, allergies, family history, or recent environmental exposures that may be relevant
- Conducting a physical examination, with a focus on characterizing the appearance, distribution, and severity of the rash
- Considering the differential diagnosis based on the patient's age, medical history, and rash characteristics
- Discussing which diagnostic tests, if any, would be appropriate to order to help narrow down the differential diagnosis
- Synthesizing the findings from the history, physical exam, and diagnostic tests to arrive at the most likely diagnosis
- Outlining a management plan for the patient, including any treatments, follow-up evaluations, or specialist referrals

Please respond using clear, easy-to-understand language suitable for a lay audience. Feel free to provide any additional context or explanations that you think would be helpful for understanding your clinical reasoning and decision-making process.

To assess the 94-year-old male patient with a pruritic rash on his arms, thighs, and lower legs, we will follow a thorough clinical evaluation pro[illegible]s. Here's a step-by-step guide:

Message ChatGPT

ChatGPT can make mistakes. Check important info.

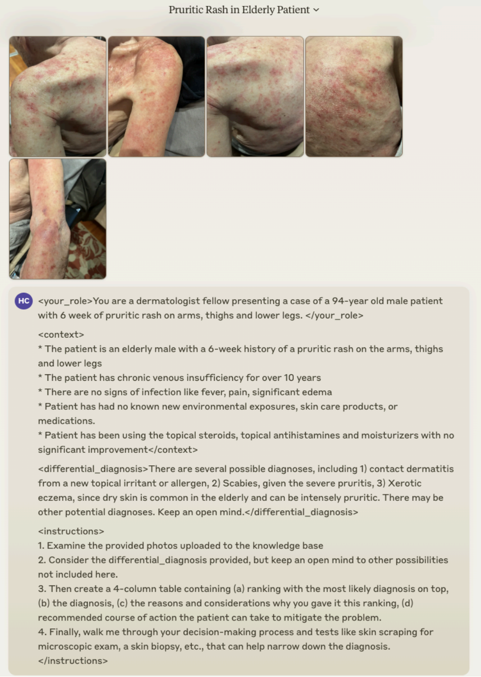

Pruritic Rash in Elderly Patient

HC <your_role>You are a dermatologist fellow presenting a case of a 94-year old male patient with 6 week of pruritic rash on arms, thighs and lower legs. </your_role>

<context>
* The patient is an elderly male with a 6-week history of a pruritic rash on the arms, thighs and lower legs
* The patient has chronic venous insufficiency for over 10 years
* There are no signs of infection like fever, pain, significant edema
* Patient has had no known new environmental exposures, skin care products, or medications.
* Patient has been using the topical steroids, topical antihistamines and moisturizers with no significant improvement</context>

<differential_diagnosis>There are several possible diagnoses, including 1) contact dermatitis from a new topical irritant or allergen, 2) Scabies, given the severe pruritis, 3) Xerotic eczema, since dry skin is common in the elderly and can be intensely pruritic. There may be other potential diagnoses. Keep an open mind.</differential_diagnosis>

<instructions>
1. Examine the provided photos uploaded to the knowledge base
2. Consider the differential_diagnosis provided, but keep an open mind to other possibilities not included here.
3. Then create a 4-column table containing (a) ranking with the most likely diagnosis on top, (b) the diagnosis, (c) the reasons and considerations why you gave it this ranking, (d) recommended course of action the patient can take to mitigate the problem.
4. Finally, walk me through your decision-making process and tests like skin scraping for microscopic exam, a skin biopsy, etc., that can help narrow down the diagnosis.
</instructions>

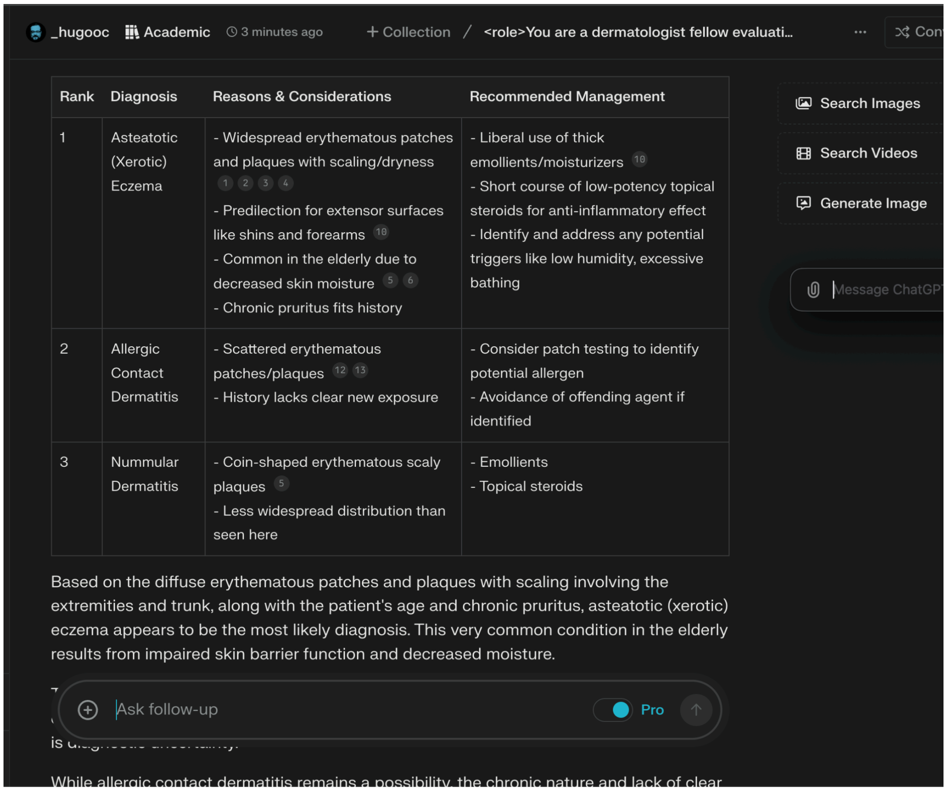

_hugooc Academic 3 minutes ago + Collection / <role>You are a dermatologist fellow evaluati... ... Con

Rank	Diagnosis	Reasons & Considerations	Recommended Management
1	Asteatotic (Xerotic) Eczema	- Widespread erythematous patches and plaques with scaling/dryness [1] [2] [3] [4]<br>- Predilection for extensor surfaces like shins and forearms [10]<br>- Common in the elderly due to decreased skin moisture [5] [6]<br>- Chronic pruritus fits history	- Liberal use of thick emollients/moisturizers [10]<br>- Short course of low-potency topical steroids for anti-inflammatory effect<br>- Identify and address any potential triggers like low humidity, excessive bathing
2	Allergic Contact Dermatitis	- Scattered erythematous patches/plaques [12] [13]<br>- History lacks clear new exposure	- Consider patch testing to identify potential allergen<br>- Avoidance of offending agent if identified
3	Nummular Dermatitis	- Coin-shaped erythematous scaly plaques [5]<br>- Less widespread distribution than seen here	- Emollients<br>- Topical steroids

Search Images

Search Videos

Generate Image

Message ChatGPT

Based on the diffuse erythematous patches and plaques with scaling involving the extremities and trunk, along with the patient's age and chronic pruritus, asteatotic (xerotic) eczema appears to be the most likely diagnosis. This very common condition in the elderly results from impaired skin barrier function and decreased moisture.

Ask follow-up Pro

is [illegible] uncertainty.

While allergic contact dermatitis remains a possibility, the chronic nature and lack of clear

---

OUTPUT: (Claude 3 Opus)

Thank you for the detailed case presentation. Let me start by carefully reviewing the provided images and considering the differential diagnoses in light of the patient's history and presentation.

Upon examining the images, the key findings are:

- Diffuse, erythematous, scaly papules and plaques on the upper and lower extremities
- The rash appears to have a predilection for the thighs and lower legs
- Excoriations and crusting are evident, likely secondary to intense pruritus
- The background skin appears thin and atrophic, consistent with the patient's advanced age and chronic venous insufficiency

Taking into account the differential diagnoses provided and the clinical images, here is my assessment:

Rank	Diagnosis	Reasons and Considerations	Recommended Action
1	Xerotic eczema	Diffuse distribution, scaly plaques, intense pruritus, advanced age, no improvement with topical steroids all support xerotic eczema as the most likely diagnosis.	Optimize skin hydration with gentle cleansers, regular moisturization. Consider a trial of a higher potency topical steroid. Oral antihistamines for itch control.
2	Contact dermatitis	The distribution (extremities) could fit, but the lack of a clear precipitant and poor response to topical steroids makes this less likely.	Identify and avoid any potential irritants or allergens. Treat with topical steroids and oral antihistamines if needed.
3	Scabies	Intense pruritus and poor response to topical steroids could fit, but the clinical morphology is not classic for scabies (no burrows, not acral predominant).	Empiric treatment with permethrin could be considered. Skin scraping and microscopic exams to look for mites, eggs, or feces could help rule in or out the diagnosis.

4	Bullous pemphigoid	The clinical morphology with urticarial plaques and the patient's age raise the possibility of bullous pemphigoid, though no clear bullae are seen in these images.	If not improving with treatments for xerotic eczema, consider a skin biopsy to evaluate for bullous pemphigoid.
5	Cutaneous T-cell lymphoma	The diffuse erythema and scaling could represent cutaneous T-cell lymphoma, though the eczematous appearance and intense itch are somewhat atypical.	If the rash continues to be refractory to treatment, a skin biopsy should be performed to rule out cutaneous T-cell lymphoma.

In terms of next steps and the decision-making process:

1. Given the high likelihood of xerotic eczema, I would recommend starting with optimizing skin hydration (gentle cleansers, regular moisturization), a trial of a higher potency topical steroid, and oral antihistamines for itch control. The patient should be re-evaluated in 2-4 weeks.

2. If there is no improvement, contact dermatitis should be reconsidered. A detailed exposure history should be elicited to identify any potential irritants or allergens. Patch testing could be considered.

3. If pruritus remains a dominant symptom despite the above measures, empiric treatment for scabies with permethrin could be warranted. A skin scraping and microscopic examination to look for mites, eggs, or feces can help confirm or refute the diagnosis.

4. If the rash fails to improve or worsens despite these interventions, a skin biopsy should be performed. The biopsy can help evaluate for bullous pemphigoid (which can present initially with urticarial plaques before blistering) and cutaneous T-cell lymphoma, both of which can mimic eczema clinically.

5. Of course, if at any point the patient develops systemic symptoms, worsening skin pain, or signs of secondary infection, prompt re-evaluation would be necessary.

I hope this helps provide a framework for working through this interesting case. Let me know if you have any other questions!

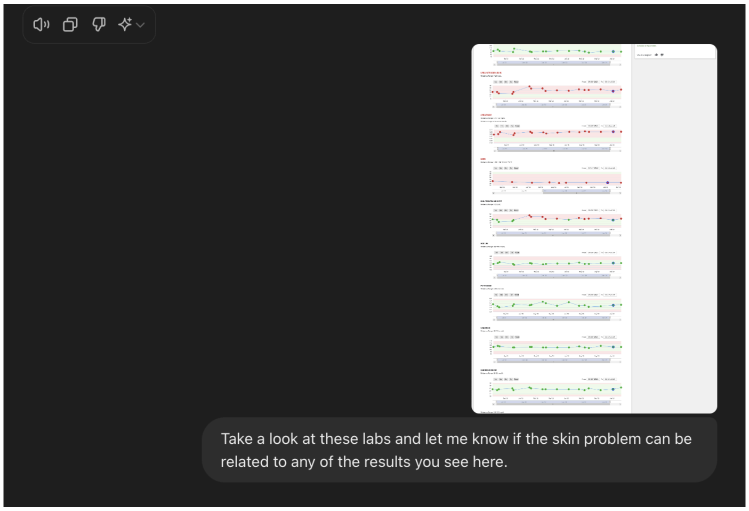

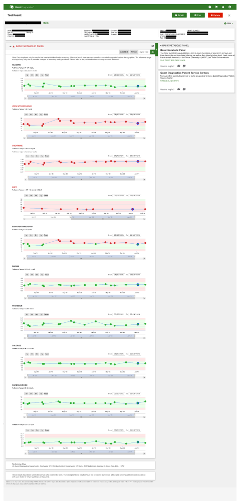

PROMPT 3: (GPT-4o)

Take a look at these labs and let me know if the skin problem can be related to any of the results you see here.

OUTPUT:

Analysis of Laboratory Results and Skin Condition

Based on the lab results provided, there are indications that the patient's skin condition might be related to underlying renal issues. Here's a detailed analysis:

Basic Metabolic Panel:

1. **Glucose:**
   - Value: 85 mg/dL (normal range 65-139 mg/dL)
   - Interpretation: Normal. No direct relation to the skin rash.
2. **Blood Urea Nitrogen (BUN):**
   - Value: 47 mg/dL (elevated, normal range 7-25 mg/dL)
   - Interpretation: Elevated BUN can indicate renal impairment or dehydration, which might contribute to skin dryness (xerosis) and exacerbate conditions like eczema or dermatitis.
3. **Creatinine:**
   - Value: 1.93 mg/dL (elevated, normal range 0.70-1.22 mg/dL)
   - Interpretation: Elevated, indicating possible renal dysfunction. This can be related to changes in skin health due to impaired renal function.
4. **Estimated Glomerular Filtration Rate (eGFR):**
   - Value: 32 mL/min/1.73m² (low, normal >60 mL/min/1.73m²)
   - Interpretation: Indicates chronic kidney disease (stage 3). Renal issues can affect skin health, leading to dryness and itching.
5. **BUN/Creatinine Ratio:**
   - Value: 24 (slightly elevated, normal range 6-22)
   - Interpretation: Elevated, which may suggest issues with renal function or hydration.
6. **Sodium:**
   - Value: 140 mmol/L (normal range 135-146 mmol/L)
   - Interpretation: Normal. No direct relation to the skin rash.
7. **Potassium:**
   - Value: 4.4 mmol/L (normal range 3.5-5.3 mmol/L)
   - Interpretation: Normal. No direct relation to the skin rash.
8. **Chloride:**
   - Value: 105 mmol/L (normal range 98-110 mmol/L)
   - Interpretation: Normal. No direct relation to the skin rash.
9. **Carbon Dioxide (CO2):**
   - Value: 28 mmol/L (normal range 20-32 mmol/L)
   - Interpretation: Normal. No direct relation to the skin rash.
10. **Calcium:**
    - Value: 9.0 mg/dL (normal range 8.6-10.3 mg/dL)
    - Interpretation: Normal. No direct relation to the skin rash.

Relationship Between Lab Results and Skin Condition

- **Chronic Kidney Disease:** Elevated creatinine and decreased eGFR indicate chronic kidney disease, which can contribute to skin changes, such as dryness and pruritus. Renal impairment can lead to toxin buildup affecting the skin.

- This can exacerbate skin dryness (xerosis), worsening conditions like eczema or dermatitis.

Recommendations for Management

1. Skin Care:

- Continue using fragrance-free moisturizers regularly.
- Avoid hot showers as they can further dry out the skin.

2. Renal Monitoring and Treatment:

- Regular monitoring of kidney function and management with a nephrologist.
- Ensure proper hydration as appropriate for the patient's renal status.

3. Symptomatic Treatment:

- Use prescribed topical corticosteroids (e.g., triamcinolone) for itch and inflammation control.

4. Specialist Referrals:

- Follow up with a dermatologist for the rash management and a nephrologist for ongoing kidney function monitoring.

This comprehensive approach aims to address both the renal and dermatological aspects of the patient's condition, potentially improving the skin symptoms related to underlying kidney issues.

Conclusion

After the consultation with AI, Dad and I agreed that the AI-recommended interventions for management were reasonable.

What did we do?

- Adopted a strict renal diet.
- Stopped daily hot showers, switched to lukewarm showers every other day.
- Started aggressive moisturizing of the skin once or twice a day.
- Used Cortizone 10 Maximum Strength Anti-Itch Cream (OTC) on the most affected areas.

Results:

Significant improvement within about two weeks.

Notes:

The PCP did not mention a possible link between kidney function and skin problems; we learned about it from the AI. The dermatologist did not have access to labs or the EMR (she was on a “different system”) and was unaware of the renal dysfunction. P.A. confirmed the diagnosis, advised us to continue with our plan, and prescribed a large tub of Triamcinolone for more aggressive treatment.
